# mitoLUHMES: An Engineered Neuronal Cell Line for the Analysis of the Motility of Mitochondria

**DOI:** 10.1007/s10571-016-0438-0

**Published:** 2016-11-10

**Authors:** Tomasz M. Stępkowski, Sylwia Męczyńska-Wielgosz, Marcin Kruszewski

**Affiliations:** 1 Centre for Radiobiology and Biological Dosimetry, Institute of Nuclear Chemistry and Technology, Dorodna16, 03-195 Warsaw, Poland; 2 Department of Molecular Biology and Translational Research, Institute of Rural Health, Jaczewskiego 2, 20-090 Lublin, Poland; 3 Department of Medical Biology and Translational Research, Faculty of Medicine, University of Information Technology and Management, ul. Sucharskiego 2, 35-225 Rzeszow, Poland

**Keywords:** Mitochondria motility, Live-cell imaging, Neuronal models, 6-OHDA, CCCP

## Abstract

Perturbations in the transport of mitochondria and their quality control in neuronal cells underlie many types of neurological pathologies, whereas systems enabling convenient analysis of mitochondria behavior in cellular models of neurodegenerative diseases are limited. In this study, we present a modified version of lund human mesencephalic cells, mitoLUHMES, expressing GFP and mitochondrially targeted DsRed2 fluorescent proteins, intended for in vitro analysis of mitochondria trafficking by real-time fluorescence microscopy. This cell line can be easily differentiated into neuronal phenotype and allows us to observe movements of single mitochondria in single cells grown in high-density cultures. We quantified the perturbations in mitochondria morphology and dynamics in cells treated with model neurotoxins: carbonyl cyanide m-chlorophenylhydrazone and 6-hydroxydopamine. For the first time we filmed the processes of fission, fusion, pausing, and reversal of mitochondria movement direction in LUHMES cells. We present a detailed analysis of mitochondria length, velocity, and frequency of movement for static, anterograde, and retrograde motile mitochondria. The observed neurotoxin treatment-mediated decreases in morphological and kinetic parameters of mitochondria provide foundation for the future studies exploiting mitoLUHMES as a new model for neurobiology.

**Electronic supplementary material:**

The online version of this article (doi:10.1007/s10571-016-0438-0) contains supplementary material, which is available to authorized users.

## Introduction

To power up their electrical activity and synapse signaling, neuronal cells need considerable amounts of ATP and Ca^2+^. Therefore, to serve their function well, post-mitotic neurons rely on cellular mitochondria pool which produce ATP as well as store and release calcium ions. Since neurons are the longest cells of human body and ATP diffusion is slow, mitochondria must be actively transported to the distant regions of the cell to support local bioenergetic requirements. This transport is realized by specialized mechanism-called microtubule sliding, driven by ATP-dependent proteins belonging to kinesin and dynein families (Hirokawa and Tanaka 2015; Barnhart 2016; Bhabha et al. 2016). Motor proteins are complexed with mitochondria by protein adaptors sensitive to various biochemical stimuli. For example, one of such protein adaptors, Milton, binds to the outer mitochondrial membrane-anchored GTPase protein Miro and this adaptor complex is sensitive to calcium ions, GTP, reactive oxygen species (ROS), and mitochondria membrane depolarization (van Spronsen et al. 2013; Loss and Stephenson 2015; Devine et al. 2016). The recent research proved that mutations in kinesin adapter proteins, together with elevated ROS and decreased activity of mitochondria, underlie the etiology of various neurological diseases, including Parkinson’s and Alzheimer’s diseases (Blesa et al. 2015; Kalia and Lang 2015).

Neuroblastoma cells, such as SH-SY5Y or BE2-M17, or pluripotent stem cells are the most widely studied in vitro models of human dopaminergic neuronal cells. However, due to their cancer origin, neuroblastoma cells differ from the phenotype of mature post-mitotic neurons to a large extent. Moreover, they need to be transcriptionally reprogrammed by addition of diverse factors into culture media, e.g., retinoic acid or phorbol ester 12-*O*-tetradecanoylphorbol-13-acetate. Differentiating of stem cells, on the other hand, allows obtaining phenotypically mature neurons but their usage is hampered by ethical issues and/or complicated and expensive protocols. Recently Lund Human Mesencephalic (LUHMES) cells appeared as a convenient model to study neurotoxicity and neurodegeneration (Schildknecht et al. 2009). LUHMES cells are embryonic mesencephalic cells, whose constant proliferation in a non-differentiated state is mediated by a switchable expression of v-MYC oncogene. After addition of tetracycline and ablation of the fibroblast growth factor from the culture medium, the cells start to differentiate into post-mitotic neuronal cells phenotypically similar to mature human neurons (Scholz et al. 2011; Stepkowski et al. 2015).

In this study, we exploited the LUHMES model and provided the statistical quantification of various parameters describing neuronal transport of mitochondria. For the first time, in the LUHMES model, we directly observed the processes of fusion, fission, and reversal of mitochondria movement direction. Hence, this work is intended as a description of a new model system; to validate its usefulness, we present the quantification of different parameters characterizing motility of mitochondria. Mean velocity, number, and length of stationary, anterograde (moving towards the neurite extension), and retrograde (moving towards the cell soma) motile mitochondria were measured in cells treated with model neurotoxins commonly used in research aimed at modeling conditions present during the development of Parkinson’s Disease: carbonyl cyanide m-chlorophenylhydrazone (CCCP) and 6-hydroxydopamine (6-OHDA). CCCP is a protonophore compound that acts at the inner mitochondria membrane. It destroys the hydrogen gradient which results in uncoupling of the oxidative chain (Heytler 1963). 6-OHDA is a neurotoxin to some extent selective for neurons expressing dopamine transporter. It acts mainly via the intercellular generation of ROS and is widely used to kill dopaminergic neurons in the animal models of Parkinson’s Disease (Storch et al. 1996). Measurements of mitochondria motility and length were possible after constructing an engineered version of LUHMES cells—the mitoLUHMES–expressing green fluorescent protein and mitochondrially targeted DsRed2 protein. Dilution of mitoLUHMES with wild-type LUHMES allows convenient live-cell imaging of single mitochondria in single cells of high-density cultures. In contrast to transient transfection of differentiated cells, usage of stable mitoLUHMES cell line enables microscopic observations of mitochondria present in the inner cells of the 3D functional neuronal network, simulating the environment of *substantia nigra* or other parts of brain. Moreover, the mitoLUHMES neuronal network can be further enhanced by addition of other cell types, such as microglia, permitting for more complex experimental design. The results will provide foundation for the future research on the factors influencing the transport of mitochondria in the LUHMES model.

## Materials and Methods

### Cell Culture

LUHMES cells were grown in monolayer in NunclonD™ cell culture flasks or Nunc Lab-Tek II microscopy live-cell imaging chambers, both coated with poly-l-ornithine (50 μg/mL) and human plasma fibronectin (1 μg/mL) (Sigma-Aldrich). Filter-sterilized poly-l-ornithine was mixed with fibronectin, poured directly to the culture flasks and chambers (1 mL per 10 cm^2^), and left overnight at 37 °C. The coating mixture was then aspirated and the vessels were dried in the laminar hood. Freshly coated chambers and flasks were used in each experiment. Undifferentiated, proliferating cells were grown in Advanced DMEM/F-12 medium (Life Technologies) supplemented with N-1 supplement (Sigma-Aldrich, N6530), basic fibroblast growth factor (bFGF) (Life Technologies) and 2 mM l-glutamine. Differentiation was conducted according to the previously established two-step protocol (Scholz et al. 2011). Briefly, when cells reached 70% confluency, medium was changed to a differentiation medium containing 1 μg/mL tetracycline instead of bFGF. On the next day (attributed as Day 2 of differentiation), cells were trypsinized and seeded to the Lab-Tek II chambers to create a monolayer of separated differentiated cells without visible clumps of unequally differentiated cells. Then the cells were further differentiated for 7 days before performing microscopic observation.

### Lentiviral Transduction and mitoLUHMES Establishment

Lentiviral plasmids were designed in the Centre for Radiobiology and Biological Dosimetry to allow expression of mitochondrially targeted DsRed2 and GFP, whereas their cloning and packaging were outsourced (CYAGEN biosciences). The mtDsRed2 lentiviral construct was built on the pLV.KD.U backbone: it contains DsRed2 protein fused with mitochondria targeting sequence under the control of a UBC (ubiquitin 3) promoter,a weak human promoter. It also contains U6 promoter that enables cloning of shRNA in future experiments. The second vector used contains GFP protein under control of a strong promoter of translation elongation factor (EF1A) gene. The detailed map of the p.LV.KD.U_mito_DsRed2_U6 viral vector used in this study is provided in supplementary material (Fig. S1). Proliferating LUHMES cells were first transduced with GFP vector and then with the DsRed2-containing particles after one week of culturing. Lentiviral particles were added to the culture medium supplemented with 4 μg/mL Polybrene (10^6^ viral particles per 1 mL). After overnight, transduction medium was removed and the cells were subcultured into 21 cm^2^ flasks for further culturing. Pure line of GFP and mtDsRed2-expressing cells was obtained by cell sorting in Becton–Dickinson Aria III sorter. Sorting was conducted after at least 10 days of culturing and several medium changes in order to avoid contamination with lentiviral particles present in the aerosol.

### Chemicals

6-Hydroxydopamine hydrochloride (Sigma-Aldrich, H4381) was dissolved in 1% ascorbic acid before every experiment and protected from light. Fresh 6-OHDA solution was added directly to culture medium to achieve the final concentration of 100 μM. CCCP (Sigma-Aldrich, C2759) was dissolved in DMSO at a concentration of 10 mM and further dissolved with cell culture medium to achieve the final concentration of 10 μM. Control cells were treated with the appropriate vehicle, 1% ascorbic acid or DMSO.

### Live-Cell Imaging

Prior to differentiation, mitoLUHMES cells were mixed with wild-type LUHMES cells in proportion of 1:200. This allowed performing microscopy imaging of the single neurons in high-density culture. Cells growing in pre-coated 8 well Lab-Tek II chambers were treated with vehicle medium or medium containing 100 μM 6-OHDA or 10 μM CCCP. Living cells were imaged by fluorescence microscopy with the use of NIKON Eclipse Ti microscope equipped in ×60 oil-immersed Plan Fluor objective (Nikon), DS-Qi1 Nikon monochrome CCD camera (1.4 numerical aperture and 1.515 refractive index), and incubator with automatic monitoring of temperature and CO_2_ levels (Oko-Lab). For confocal images, we used Nikon A1 confocal; and 488 argon laser was used to excite GFP emission. For 3D imaging, a water immersed ×60 NIKON objective was used.

### Time-Lapse Recording and Digital Compression

For statistical analysis, we recorded 30 min long movies. First, just one frame in GFP channel was captured to visualize the neurites, but to avoid unnecessary phototoxicity and a delay caused by the filter change, the subsequent photos were taken only in DsRed2 channel. We used the same fluorescent lamp settings, exposure time, and resolution for every film recorded. The differences in intensity of fluorescence between particular neurons were compensated only by changing the gain of the CCD output. Thus all recorded cells were exposed to the same amount of light for the identical period of time to standardize phototoxicity that might interfere with the measurements. The following camera parameters were used: GFP channel: resolution 1280 × 1024 (no binning mode), exposure time 200 ms, analog gain up to ×2; DsRed2 channel: resolution 640 × 512 (no binning mode), exposure time 300 ms, analog gain in the range from ×1 to ×9.6. Due to hardware lag, the acquired interval between frames ranged from 500 ms to 600 ms. Original raw data files (NIS ELEMENTS software) were exported into avi format without any compression. The obtained avi files were subsequently compressed with Windows Movie Maker into a mpeg-4 H.264 format—29 frames/s. The movies that were not used for statistical analysis, but for visualization of mitochondria fission, fusion, and reversal of motion direction were recorded with various frame rates, and original time is indicated inside every movie. Further details are provided in movie legends.

### Evaluation of CCCP and 6-OHDA Cytotoxicity (Impact on Metabolic Mitochondrial activity)

The impact of CCCP and 6-OHDA on metabolic activity of LUHMES cells was measured with resazurin assay. In brief, LUHMES cells that had grown in the differentiation medium for one day were seeded in 24-well plates (NunclonΔT surface) at a density of 2 × 10^5^ cells/well in 500 µL of culture medium and differentiated for subsequent 6 days. Three independent experiments with four technical replicates in each were conducted. After differentiating the cells, we treated them, for 4 and 7 h, with increasing concentration of CCCP (2, 10, 50 µM) and 6-OHDA (40, 100, 250 µM). After incubation with tested compounds, 50 µL of resazurin dye solution (Sigma-Aldrich) was introduced for the subsequent two hours of incubation. Cells were lysed with 250 µL of 3% SDS solution (3% SDS in PBS) and fluorescence (Ex = 560 nm, Em = 590 nm) of cell lysates was measured after 10 min of gentle shaking in plate reader spectrophotometer Infinite M200 (Tecan, Austria).

### Statistical Analysis

For cytotoxicity analysis, three independent experiments were conducted for each toxicity point. Difference between samples and controls was calculated with Kruskal–Wallis One-Way Analysis of Variance on Ranks (ANOVA) followed by post hoc Dunnett’s method. Analysis was performed with GraphPad Prism 5.0 software (Graphpad Software Inc., USA). Differences were considered statistically significant when the *p* value was less than 0.05.

The recordings, of mitochondria motility in toxin-treated cells, were performed in three independent biological replicates, whereas those of control cells, were performed in five independent biological replicates (recordings of the new batch of differentiated cells). The biological replicates consisted of two 30-min long recordings (technical replicates performed at the same day on the same cells grown on the same coverglass but in the different chambers) or up to five, if one or both of the recorded cells in a particular time point and treatment did not contain any retro or anterograde motile mitochondria. As well treated and control cells were recorded at the same day and from the same source of cells grown in a 8-well chambered coverglass. Statistical calculation was performed in Microsoft Excel. Statistical significance was estimated by two-sample Student’s *t*-test assuming equal variation.

## Results

### Differentiated mitoLUHMES Display Morphological Features of Mature Neurons and Enable Performing High-Quality Visualization of the Motility of Mitochondria

**Fig. 1:**
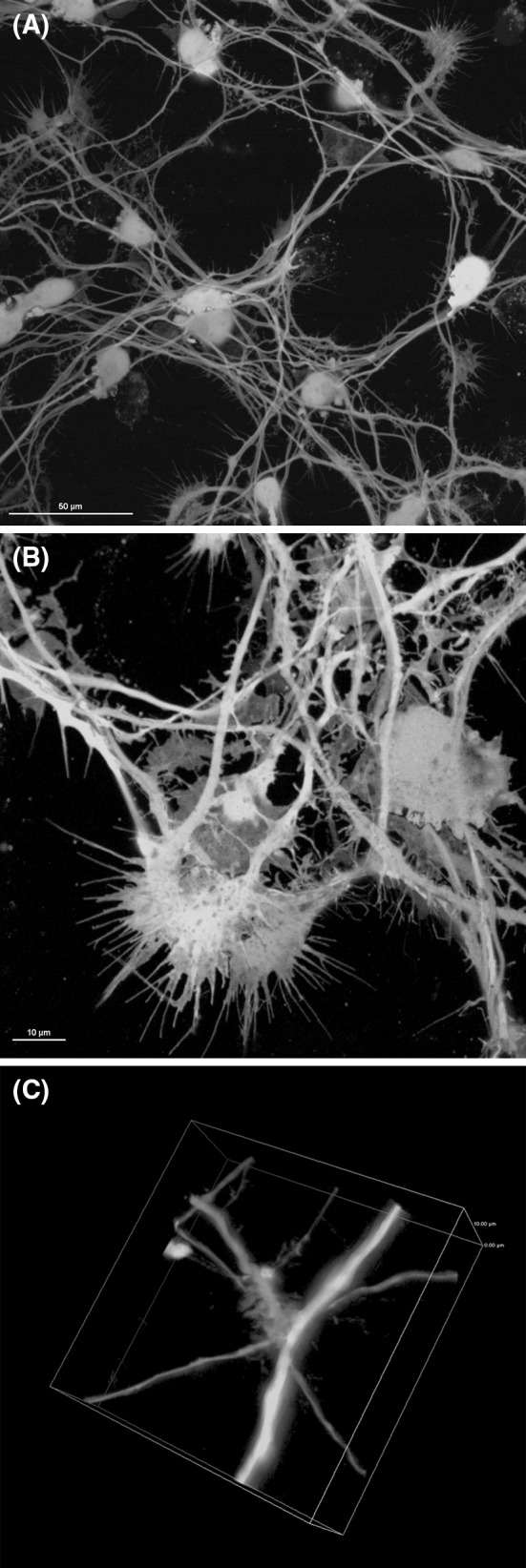
The morphology of differentiated LUHMES cells. Confocal image of: **a** highly branched GFP-expressing LUHMES neuronal culture (magnification ×60). **b** Growth cones in GFP-expressing differentiated LUHMES cells. **c** Neurite branching and dendritic spikes in GFP-expressing differentiated LUHMES

**Fig. 2:**
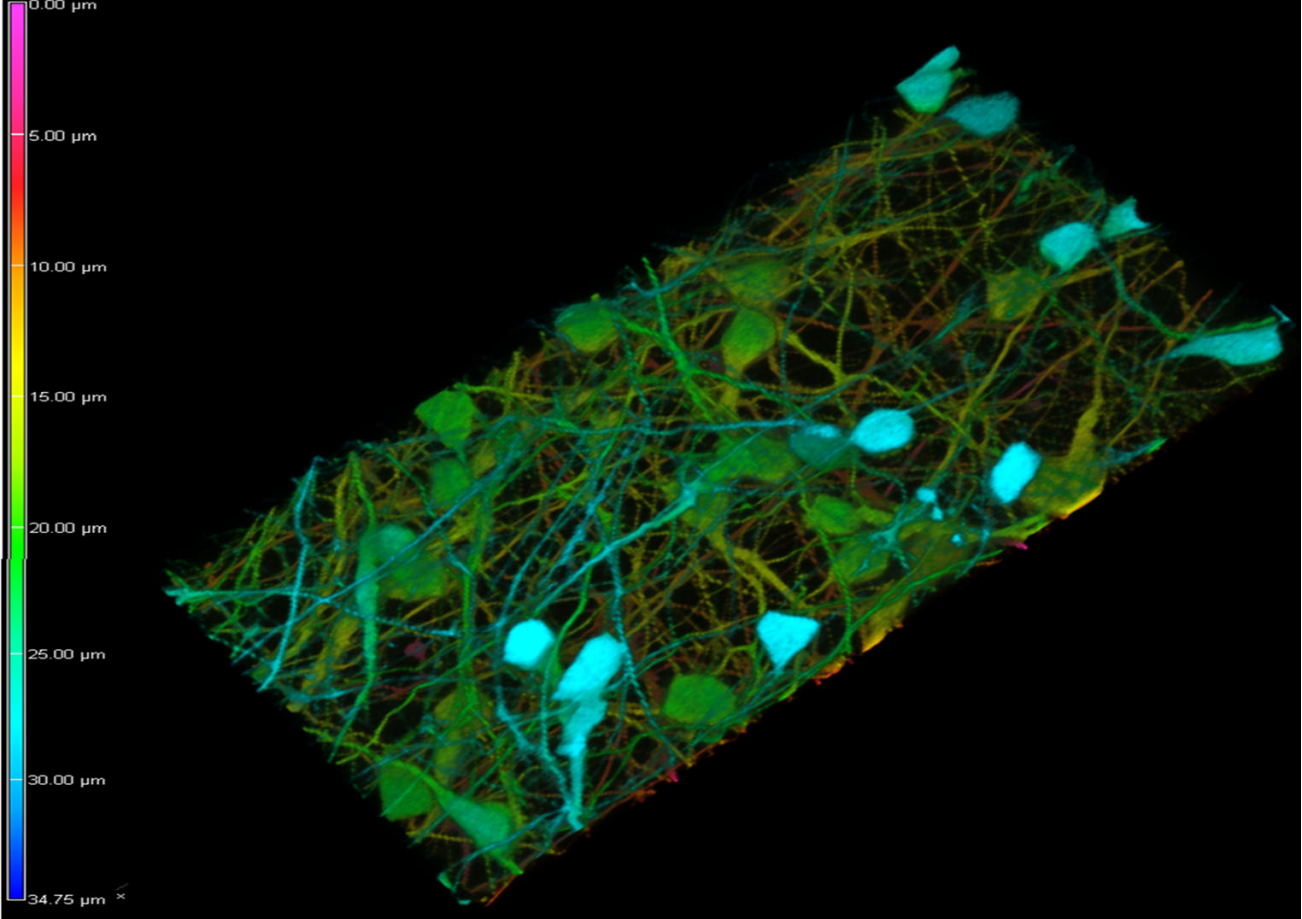
The 3d reconstruction of the high-density neuronal network of differentiated GFP-expressing LUHMES cells mixed with wild-type LUHMES at proportions 1:20. The depth of the network reaches 30–35 μm. The depth color coding is displayed on the left border of the image

**Fig. 3:**
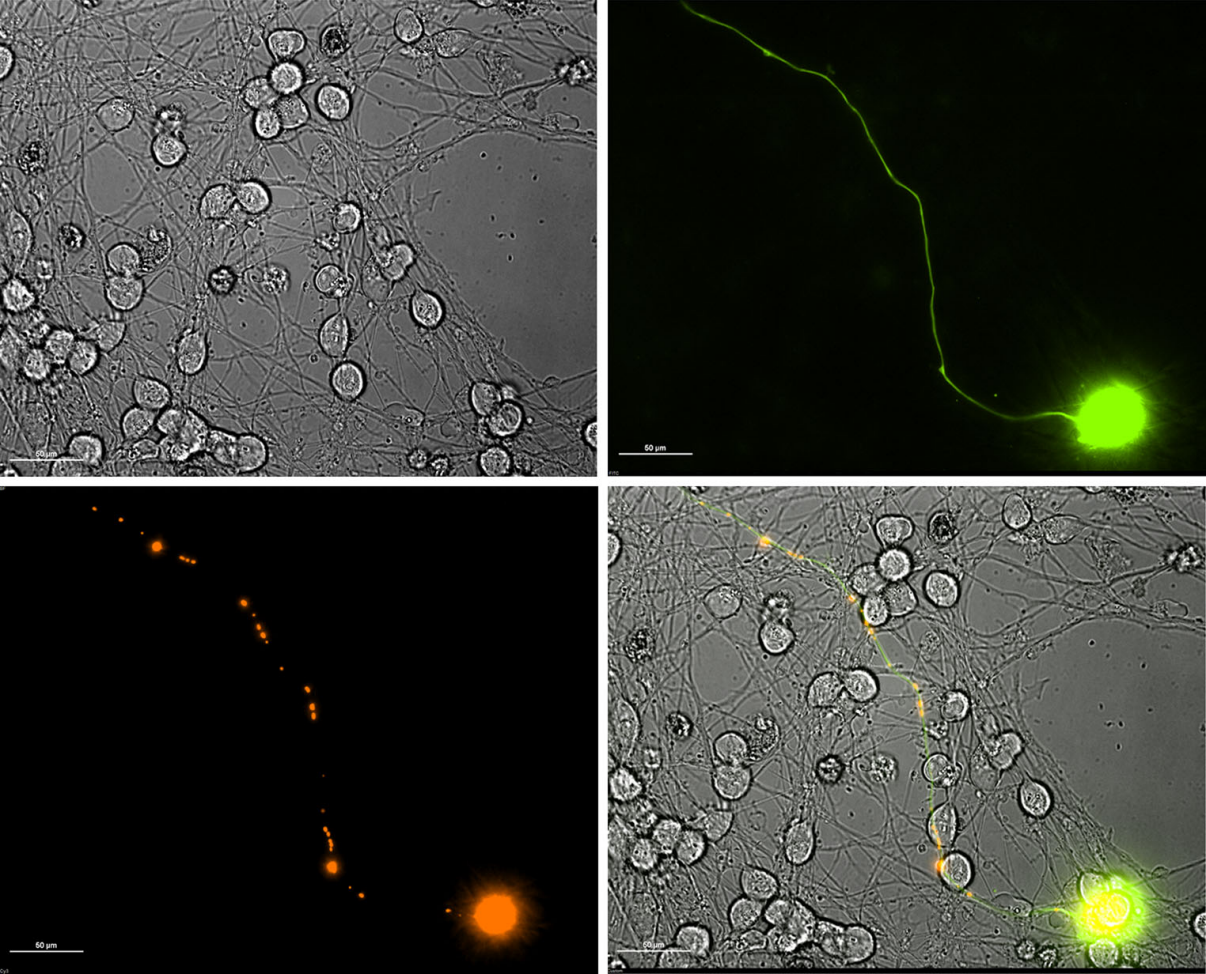
mitoDsRed2 and GFP-expressing mitoLUHMES cells mixed with wild-type LUHMES cells at proportion 1:200 allow visualization of individual mitochondria in a single neurite. The cells in this sample were treated with 6-OHDA for 5.5 h and therefore a fragmented circular mitochondria are clearly visible

Using the protocol by Scholz et al. (2011), we successfully differentiated the cells and obtained highly interconnected neuronal network with visible neuronal features, such as very long neurites (up to 1000 μm), growth cones, dendritic spikes, and extensive neurite branching (Fig. 1). The Z depth of this network achieved around 30 μm for high-density culture (Fig. 2). Culturing mitoLUHMES with wild-type LUHMES in proportion 1:200 allowed us to visualize and record the motility of mitochondria in single neurons (Fig. 3). Mitochondrial motility recorded in pure culture of mitoLUHMES grown in low density is presented in supplementary movie 1 (Online Resource MOESM3).

### The Observation of Fission and Fusion of Mitochondria in the mitoLUHMES Cell Line

For the first time in the LUHMES cells, we recorded the time-lapse movies presenting the processes of mitochondria fission/and fusion in neurites. This was possible with the use of a sensitive Nikon DS-Qi1 CCD camera. We observed the fissions of motile as well as stationary mitochondria. Fusions were also clearly visible and occurred more frequently between stationary and motile mitochondria than between two mitochondria being in motion (Online Resources MOESM4 and MOESM5).

We observed that motile mitochondria often stop at places enriched in stationary mitochondria and that this stop is often followed by an increase of velocity and/or reversal of motion direction of mitochondria. Although it is difficult to observe, one might suggest that during this stop, a quick time fusion (a kiss-and-run type) and/or exchange/binding of kinesin/dynamin or their adaptor proteins occurs (Online Resource MOESM6).

### Transport of Mitochondria in Neurites is Discontinuous in LUHMES Cells

By performing high-speed time-lapse-observation, we found out that the mitochondria motion in LUHMES cells was discontinuous and not uniform. It was characterized by subsequent events of motility and pausing. The pauses had a duration of 1–20 s, usually followed by a continuous mitochondria motion. As a result, a considerable difference in velocity was observed between continuous motion (short-path velocity) and long-path velocity, when discontinuous motion (accounting pauses) was measured (Online Resource MOESM7). Due to this ambiguity, the analysis of retrograde and anterograde mitochondria velocities presented in the following paragraphs is based on short-path velocity comparison.

### Reversal of the Motion Direction of Mitochondria

Our time-lapse observation revealed that individual mitochondria moving anterograde and retrograde can steadily change their direction: anterograde mitochondria turn back and start their journey towards the cell soma, whereas retrograde mitochondria turn back and start their way towards the neurite extension (Online Resource MOESM8). The events of direction turning are preceded by pausing and/or fusion events, suggesting the need of the rearrangement of the molecular motor complex machinery.

### The Impact of CCCP and 6-OHDA Treatment on Mitochondrial Metabolic Activity

**Fig. 4:**
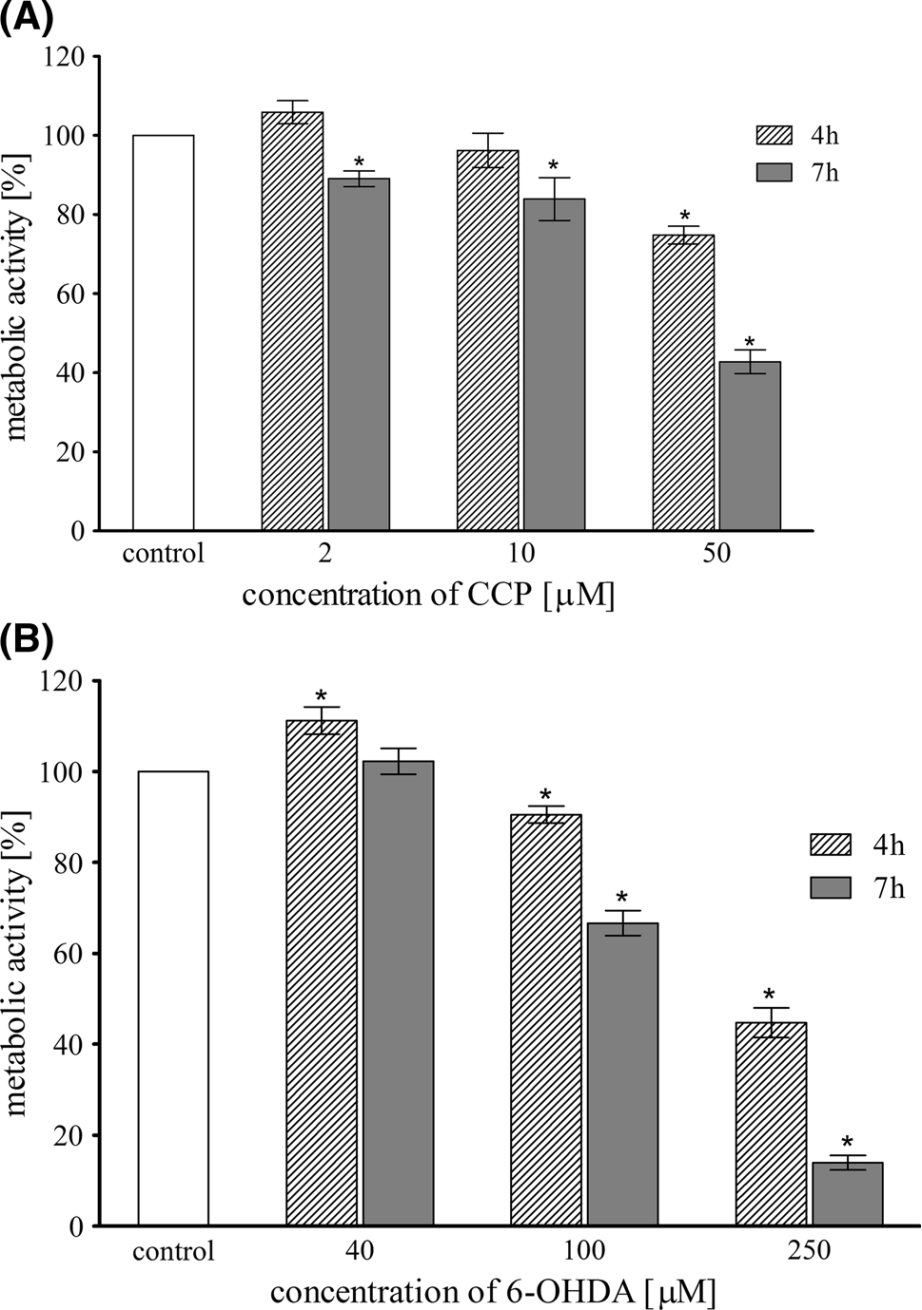
Metabolic activity (resazurin assay) of LUHMES cells treated with different concentrations of CCCP (**a**) and 6-OHDA (**b**) for 4 and 7 h. Data are expressed as a percent of control, mean ± SD from three independent experiments (*asterisk* denotes statistically significant difference from vehicle-treated control, *p* < 0.05)

The in vitro cytotoxicity (mitochondrial metabolic activity) of CCCP and 6-OHDA was investigated in LUHMES cells as a function of concentration. Two incubation times were used: 4 and 7 h. The metabolic activity was assessed by colorimetric resazurin assay in comparison to vehicle-treated cells (Fig. 4). Both compounds significantly reduced mitochondria metabolic activity in a dose-dependent manner. Lower concentrations of examined compounds (2, 10 μM CCCP and 40, 100 μM 6-OHDA, respectively) were moderately toxic in comparison to the highest concentrations used: CCCP (50 μM) reduced the metabolic activity of LUHMES cells after 7 h incubation to 40% of control value, whereas 6-OHDA (250 μM) reduced the metabolic activity of LUHMES cells up to 14% of control value. The results proved that 10 μM CCCP and 100 μM 6-OHDA show moderate cytotoxicity much above the IC_50_ and those concentrations were used for all the following experiments.

### Mitochondria in Cells Treated with 6-OHDA and CCCP are Shorter

In order to investigate the effects of neurotoxins on shape and motility of mitochondria, cells were treated with 10 μM CCCP for 2, 4, and 5 h and 100 μM 6-OHDA for 2.5, 4.5, or 5.5 h. To evaluate the degree of mitochondrial fragmentation after neurotoxin treatment, we measured the length of mitochondrial major axis.

**Fig. 5:**
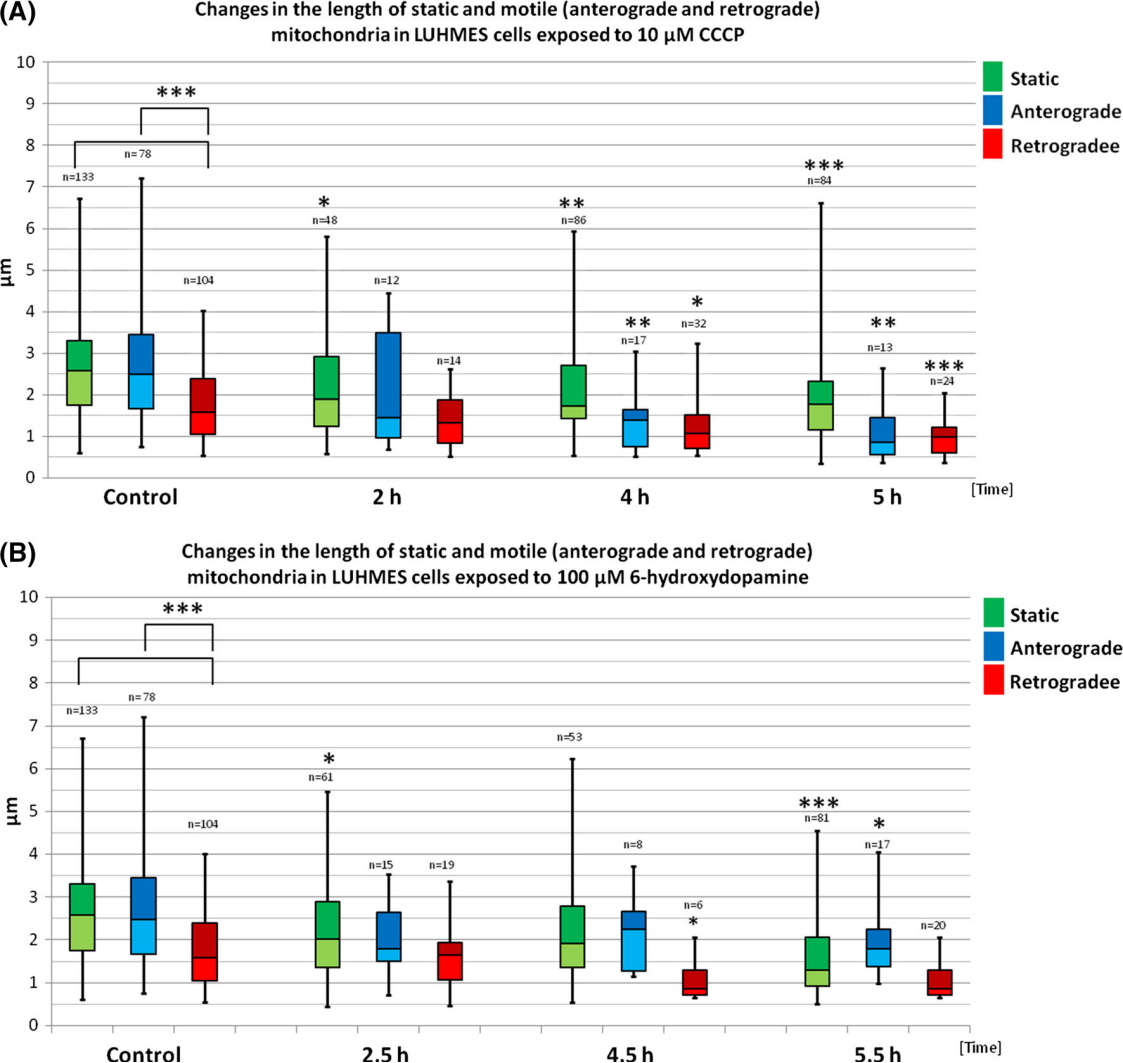
The length of static (*green*), anterograde (*blue*), and retrograde (*red*) mitochondria treated with CCCP (**a**) or 6-OHDA (**b**) in indicated time points. The results are presented as *box-whiskers* plots. Median values are shown as *boxes borderline*. The box top–bottom values are defined by the 25th and 75th percentile. The ends of the *whiskers* represent the minimum and maximum values. The *n* value represents the number of measured mitochondria. The statistical significance between the static, retrograde, and anterograde groups and corresponding controls is shown as *asterisk*. *p < 0.05. **p < 0.005; ***p < 0.0005

In vehicle-treated cells mitochondria length ranged from around 0.5 μm to around 7.0 μm. Interestingly, the retrograde mitochondria were shorter than stationary and anterograde (1.75 ± 0.85 vs. 2.7 ± 1.26 and 2.79 ± 1.53 μm respectively; *p* < 0.001). This difference became less pronounced or disappeared in cells treated with the tested neurotoxins. CCCP treatment resulted in the appearance of a population of small, fragmented stationary mitochondria, but distribution of the longest stationary mitochondria remained unchanged (Figs. 5, S2). Conversely, only small mitochondria (shorter than 3 μm) were observed in the anterograde group after 4 h and 5 h of CCCP treatment, suggesting that the largest anterograde motile mitochondria might have been stalled and joined the stationary mitochondria pool. As compared with 6-OHDA, CCCP treatment resulted in more pronounced mitochondria fragmentation. Both motile and stationary mitochondria were shorter in the treated cells and the decrease in mitochondria length was time dependent.

Similarly to CCCP, 6-OHDA treatment also resulted in the decrease in mitochondria length although the observed decreases were less pronounced and not as time-dependant as those observed in CCCP-treated cells. Moreover, conversely to CCCP-treated cells, only slight decreases in the length of retrograde moving mitochondria were observed in 6-OHDA-treated cells only after 4.5 h.

**Fig. 6:**
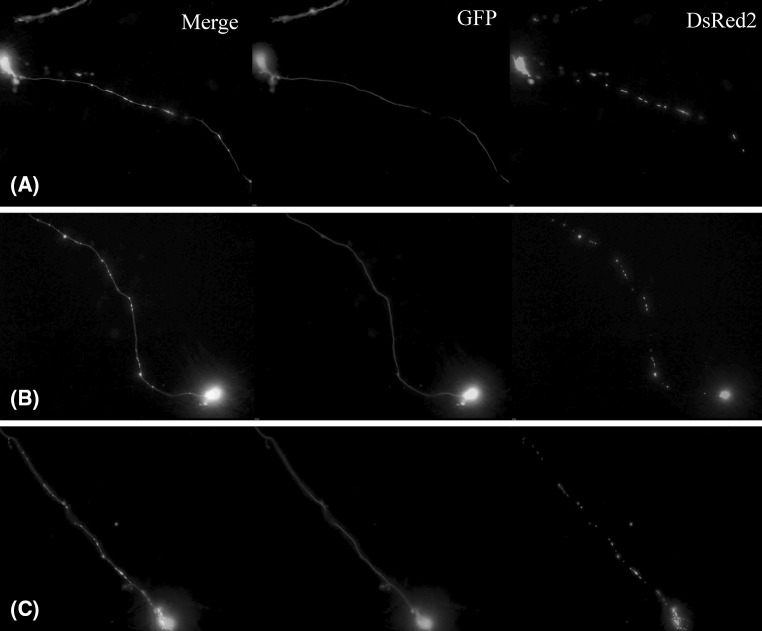
The morphology of mitochondria in mitoLUHMES cells grown with wt LUHMES at proportion of 1:200 **a** Vehicle-treated control cells (0.1% DMSO 5 h); **b** 5.5 h of incubation with 100 μM 6-OHDA; **c** 5 h of incubation with 10 μM CCCP

The microscopic images showing the most pronounced fragmentation taking place after 5 h of treatment with CCCP and 5.5 h of treatment with 6-OHDA are shown in Fig. 6.

### CCCP But not 6-OHDA Treatment Resulted in the Decrease in the Velocity of Mitochondria

**Fig. 7:**
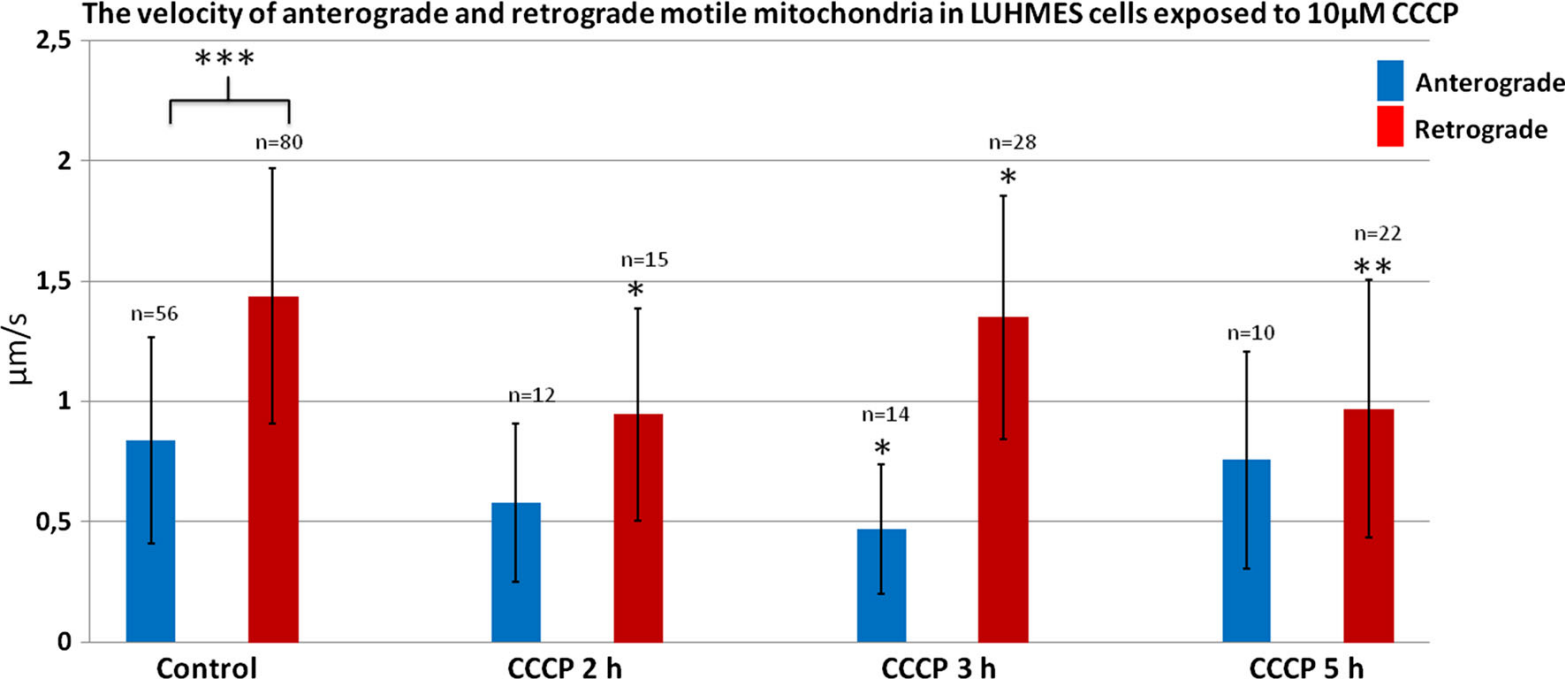
The mean velocity of anterograde and retrograde motile mitochondria in cells treated with CCCP for 2, 3 and 5 h. The *error bars* represent ±standard deviation. The statistical significance of the difference between retrograde mitochondria velocity in control and CCCP-treated cells is marked by *asterisk*: *p < 0.05. **p < 0.005; ***p < 0.0005

The velocity of motile mitochondria was calculated from the length of tracks of continuous motion. As already mentioned, the “short-path” velocity was higher than the “long-path velocity” (see paragraph 2, Online Resource MOESM7). Retrograde mitochondria moved faster than anterograde (1.44 ± 0.53 vs. 0.84 ± 0.43 μm/s, respectively; *p* < 0.01). Interestingly treatment with CCCP, and not 6-OHDA (data not shown), affected mitochondria velocity, especially the retrograde ones. The velocity of retrograde mitochondria motility decreased after 2, 3 and 5 h of treatment to 0.95 ± 0.44 μm/s, (*p* < 0.01); 1.35 ± 0.5 μm/s, (*p* < 0.05), and 0.97 ± 0.53 μm/s (*p* < 0.001), respectively (Fig. 7). We also observed the tendency for a decrease in velocity of the anterograde mitochondria, but found statistical significance for this decrease only after 3 h of treatment. Noteworthy, the frequency of transport events observed in CCCP-treated group was much lower than in controls (see below).

### CCCP and 6-OHDA Treatment Decreased the Frequency of Events of Retro- and Anterograde Transport of Mitochondria

**Fig. 8:**
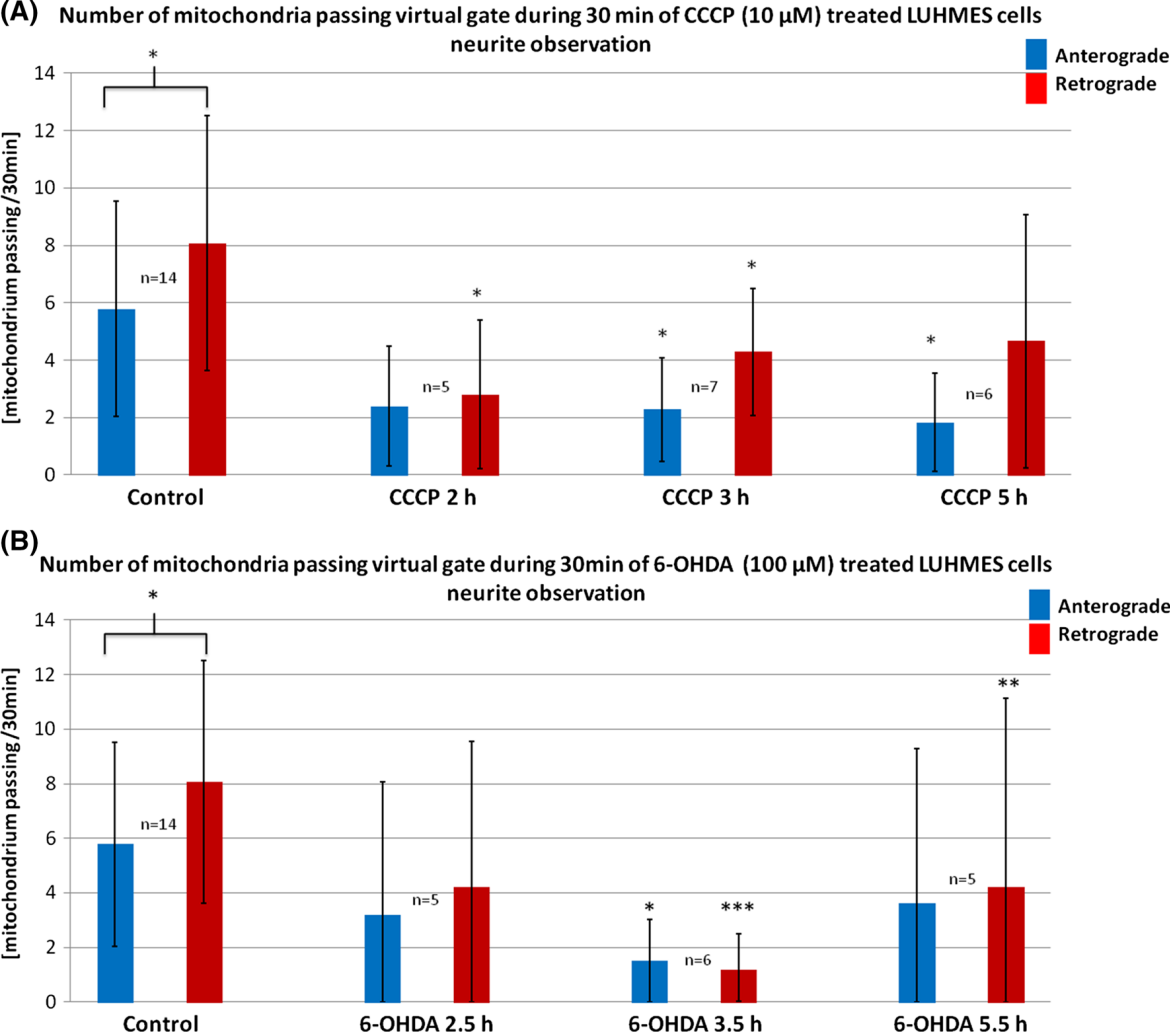
Mean frequency of events of transport of mitochondria in differentiated LUHMES cells treated with **a** CCCP and **b** 6-OHDA in indicated time points. The *error bars* represent ±standard deviation. The statistical significance between treated cells and control is denoted by *asterisk* *p < 0.05. **p < 0.005; ***p < 0.0005. The *n* indicates number of replicates (cells analyzed)

**Fig. 9:**
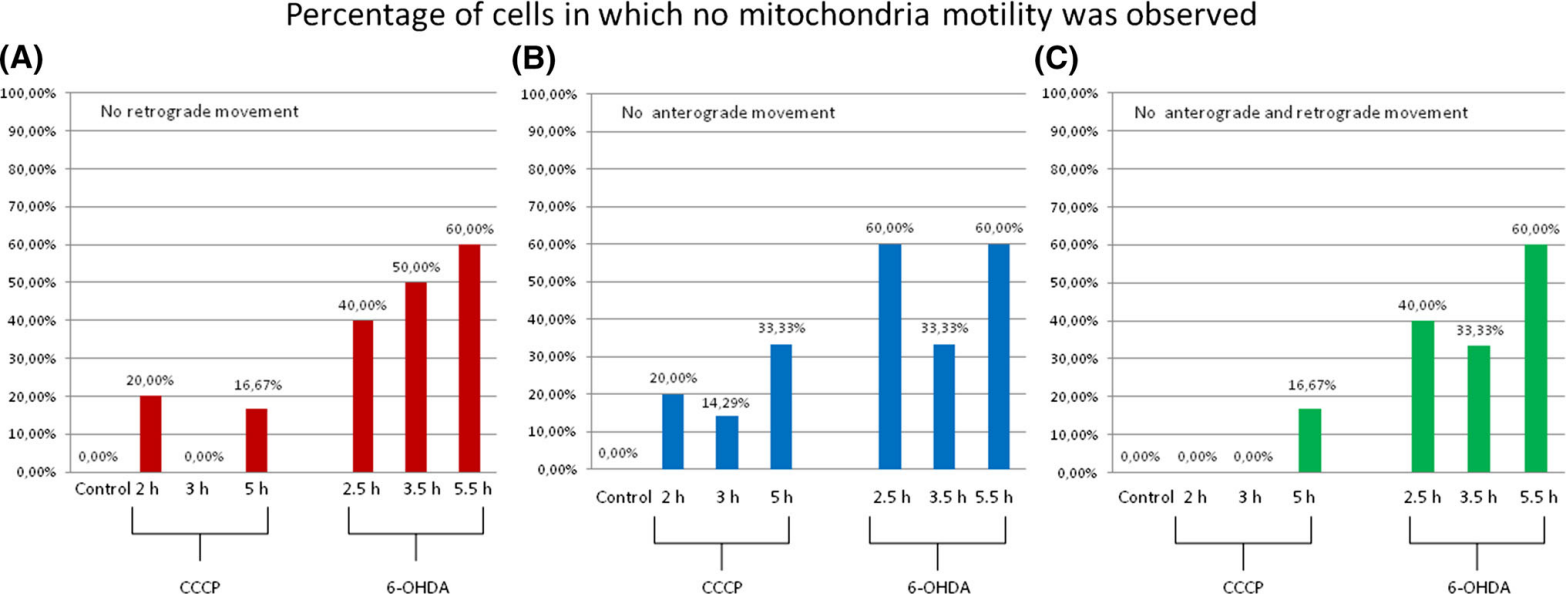
Percentage of mitoLUHMES cells in which mitochondria motility was stopped after treatment with indicated concentrations of CCCP and 6-OHDA. The left (*red*) panel—percentage of cells in which at least retrograde movement was not present; the inner panel (*blue*)—the percentage of cells in which at least anterograde movement was not present; the right panel (*green*)—the percentage of cells in which we did not observe any motile mitochondria

The events of retrograde transport of mitochondria were more frequent than the events of anterograde transport [8.07 ± 4.45 (events/30 min) vs. 5.78 ± 3.75 (events/30 min) respectively; *p* < 0.05]. The treatment with CCCP and 6-OHDA led to decrease or total inhibition of the transport of mitochondria (Figs. 7, 8, 9). In the 40% up to 60% of cells treated with 6-OHDA only stalled mitochondria were observed. Noteworthy, the velocity of the mitochondria that remained motile after 6-OHDA treatment remained unchanged (data not shown). Conversely, in CCCP-treated cells more motile mitochondria were observed, but their velocity was lower compared with the control cells mitochondria (Figs. 7, 9).

## Discussion

We engineered human neuronal LUHMES cells to create a very convenient model—the mitoLUHMES—to study various biological processes impacting the morphology and dynamics of mitochondria. Taking advantage of the mitoLUHMES utility, we captured good-quality live-cell recordings of mitochondrial motility in differentiated human neurons and the statistical description of the observed events. The short exposure times (300 ms) allowed us to visualize fast processes, such as the discontinuity in the motion of mitochondria. Moreover, despite relatively long period of recording (30 min), we did not observe any visual signs of phototoxicity. Noteworthy, we also did not observe any adverse effect of mitoDsRed2 transduction, although, similarly to DsRed1 fluorescent protein, mitoDsRed2 probably forms potentially biologically active tetramers. Noteworthy, the DsRed2 oligomers do not further aggregate, thus would not interfere with mitochondrial membrane proteins (Yarbrough et al. 2001). Moreover, in mitoLUHMES cells, DsRed2 expression is driven by a weak UBC promoter and the protein should not reach the levels possibly capable of interfering with mitochondrial functions (Fig. S1). Indeed, we did not find any differences in the morphology of mitochondria between mitoLUHMES cells and *MitoTracker* stained wt LUHMES. Moreover, the cellular division times were not altered in proliferating mitoLUHMES cells (data not shown).

For the first time in LUHMES cells, we recorded the processes of fusion, fission, and reversal of the motion direction of mitochondria, proving that mitoLUHMES could be an excellent model to study mitochondrial behavior. The molecular foundations of fission/fusion cycles of mitochondria are relatively well deciphered. In comparison, the phenomenon of reversal of the motion direction of mitochondria still remains elusive, although was described earlier (Ligon and Steward 2000; Bertholet et al. 2016). From our recordings (Online Resource MOESM8) it is clearly visible that the event of reversal of the motion direction is not preceded by any interaction with stationary mitochondria. This observation is in line with Ligon and Steward (2000), who described a “saltatory motion” of mitochondria, as well as their direction turning in cultured hippocampal neurons (Ligon and Steward 2000).

Our analysis of the statistical distribution of mitochondria length revealed that in control cells motile and stalled mitochondria can be attributed to different subgroups on the basis of their size. It is believed that size of mitochondria corresponds to its bioenergetic capacity. Moreover, under certain stress conditions, either fusion or fission of mitochondria can be beneficial to keep them functioning. Fusion enables mixing of mitochondria content between damaged and intact ones and therefore, may protect against the loss of function related to mtDNA mutation or protein oxidation. Mitochondrial fission on the other hand, may have a protective role by allowing efficient “recycling” of mitochondrial content and/or removal of damaged mitochondria in a process of mitophagy (Westermann 2012). In line with the role of CCCP and 6-OHDA in induction of mitophagy, we observed a large fraction of small mitochondria in every treated group (Solesio et al. 2012; Sargsyan et al. 2015). Noteworthy, the percentage of large immotile mitochondria in the stationary mitochondria pool remained on similar levels. We hypothesize that it can be explained by stagnation of anterograde mitochondria accompanied by fission of large stationary mitochondria or conversely, by a fission and stagnation of anterograde mitochondria without fission of the large stationary pool. It is believed that during mitophagy, mitochondria must first undergo fission and retrograde transport to the cell soma abundant of lysosomes (Lee et al. 2011; Wong and Holzbaur 2015). In contrary, Ashrafi et al.(2014) showed that mitophagy in neuronal axons may occur distally from the cell soma (Ashrafi et al. 2014). This is in line with our observation of decrease in the number of both anterograde and retrograde transport events after CCCP and 6-OHDA treatment. Moreover, we found that neurotoxin treatment resulted in the appearance of shorter mitochondria that may be either a substrate for mitophagy or a functional small mitochondria created from fission of a larger damaged mitochondria. This needs to be investigated.

The similar system enabling live-cell imaging of mitochondria in LUHMES cells was presented by Schildknecht et al. (2009) who studied the motility of TurboRFP-targeted mitochondria in cells exposed to a Parkinson’s Disease model toxin—MPP^+^ (Schildknecht et al. 2009). Similar to us, Schildknecht et al. (2009) observed a decrease in the velocity of mitochondria in cells treated with toxin. Although, neither treatment time nor mechanism of action of MPP^+^ is similar to CCCP and 6-OHDA, the toxins end-effects are similar: generation of ROS and ATP depletion (Gonzalez-Polo et al. 2004). Both these processes could influence the function of motor proteins. Schildknecht et al. (2009) did not study the velocity of the retrograde and anterograde mitochondria alone, but reported that the total velocity was quite evenly distributed (around 2.3 μm/s). This value is higher than the values measured by us (1.44 ± 0.53 and 0.84 ± 0.43 μm/s for retro- and anterograde mitochondria, respectively). This discrepancy may be caused by different times and strategies of measurement. We recorded 30-min fast time-lapse movies in ×60 magnification, whereas Schildknecht et al. (2009) recorded 4 min in ×40 magnification and performed semi-automatic kymograph quantification. Thus, it is possible that due to the short time of observation, Schildknecht et al. (2009) quantified only the fastest motile mitochondria, or our manual tracking better represented the actual, ellipsoid-shaped mitochondria tracks compared to straight line diagonals generated by kymograph. Despite different velocity values observed by us, the average number of mitochondria per 100 μm of neurite and toxin-mediated mitochondria stagnation was similar to those observed by Schildknecht et al. (2009). Similarly, those authors also noticed a slight increase in retrograde motile mitochondria.

The treatment with 6-OHDA, more effectively than CCCP treatment, inhibited the events of transport of mitochondria. On the other hand, 6-OHDA compared with CCCP did not decrease the velocity of mitochondria that remained motile. Similar to us, Lu et al. observed that 6-OHDA led to a significant decrease in the number of motile mitochondria, but had no effect on the velocity of mitochondria that remained motile in axons from mouse midbrain cells cultured in vitro (Lu et al. 2014). The authors also found out that free radical scavengers rescued the stalled mitochondria phenotype, suggesting that appearance of ROS is a key factor modulating mitochondria motility after 6-OHDA intoxication. It is however not clear why 6-OHDA did not impact the velocity of mitochondria that remained motile after treatment. In case of CCCP, its impact on inhibition of mitochondria velocity could be explained simply by the depletion of ATP pool and hampered motor proteins functionality and/or by the CCCP-induced increase in free Ca^2+^ ion pool, known to inhibit mitochondria motility in mechanism related to molecular motor adapter Ca^2+^-binding protein Miro (Guo et al. 2005; Fransson et al. 2006; Devine et al. 2016).

In conclusion, we developed and validated a convenient model that can be easily adopted to decipher various unsolved problems related to morphology and motility of mitochondria in neuronal cells. The mitoLUHMES model allows obtaining cells phenotypically similar to mature dopaminergic or cholinergic neurons and has considerable advantages over the other commonly used in vitro neuronal models such as SH-SY5Y neuroblastoma cells. Moreover, it allows detailed observations of mitochondria morphology and dynamics in 3D environment and/or studying those processes during neuronal differentiation. Mixing with wild-type cells, on the other hand allows for precise observation and measurement of neurites of single cell grown in high-density culture.

Mitochondria are now widely accepted to underlie the etiology of widespread neurodegenerative diseases, such as Parkinson’s and Alzheimer’s disease. Therefore, we hope that the presented system will become a useful tool to study the dynamic mitochondria behavior in neurons. We also hope that the open access policy would encourage other scientists to benefit from our movies concerning mitochondria biology in their presentations and lectures for educational purposes.

## Electronic Supplementary Material

Below is the link to the electronic supplementary material.

Supplementary Fig. S1. The map of the lentiviral plasmid used for mitoDsRed2 transduction (TIFF 55 kb)

Supplementary Fig. S2. The number of stationary mitochondria per 100 μm neurite length in cells treated with CCCP (A) or 6-OHDA (B). The number of stationary mitochondria was calculated from 20-40 μm in focus fragments of neurites and results were extrapolated. The statistical significance between treated cells and control is denoted by asterisk * - *p* < *0.05*. ** - *p* < *0.005*; *** - *p* < *0.0005* (TIFF 112 kb)

Supplementary Movie 1. Mitochondria motility in neurites of differentiated LUHMES (Lund Human Mesencephalic) neuronal cells. Cells were modified to express mitochondrially targeted DsRed2 fluorescent protein and GFP. Mitochondria remarkably differ in motility, with velocity reaching 2.7 μm/s for the fastest mitochondria moving towards the cell soma (retrograde movement). One can also observe the fusion and fission cycles of mitochondria. Time lapse video: 10 min of real time compressed to ~1 min, in total 804 frames were taken for mtDsRed2, 3 frames were taken for GFP. The camera exposure time for DsRed2 was set to 150 ms, however the total average time between the recorded frames was 700 ms due to the technical delay caused by computer hardware (MP4 19230 kb)

Supplementary Movie 2. Time lapse video showing mitochondria fission in neurite of differentiated mitoLUHMES cell. The real time is indicated in the top (MP4 394 kb)

Supplementary Movie 3. Time lapse video showing mitochondria fission and fusion events in neurite of differentiated mitoLUHMES cell. The real time is indicated in the top (MP4 3524 kb)

Supplementary Movie 4. Mitochondria fusion, fission and reversal of motion direction in neurite of differentiated mitoLUHMES cell. The real time is indicated in the top. The retro and anterograde direction is indicated by red arrows (MP4 1012 kb)

Supplementary Movie 5. The small ~ 1 μm long mitochondrion moving in retrograde direction display pattern of discontinuous motility characterized by phases of fast velocity motion and pausing (MP4 203 kb)

Supplementary Movie 6. The event of reversal of mitochondria motion direction in neurite of differentiated LUHMES cells (MP4 19124 kb)
