# Supplementary figures and images for: mitoLUHMES: An Engineered Neuronal Cell Line for the Analysis of the Motility of Mitochondria
Source: Cell Mol Neurobiol. 2016 Nov 10;37(6):1055–66. doi: 10.1007/s10571-016-0438-0 (PMC5494036; doi:10.1007/s10571-016-0438-0)

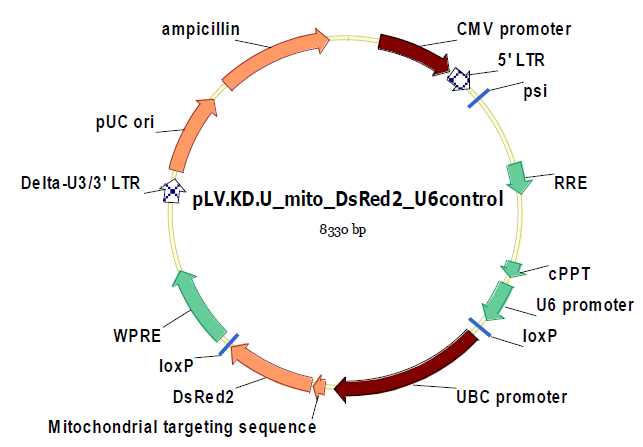

Supplement: Supplementary file 1 — Supplementary Fig. S1. The map of the lentiviral plasmid used for mitoDsRed2 transduction (TIFF 55 kb) [file 10571_2016_438_MOESM1_ESM.tif]

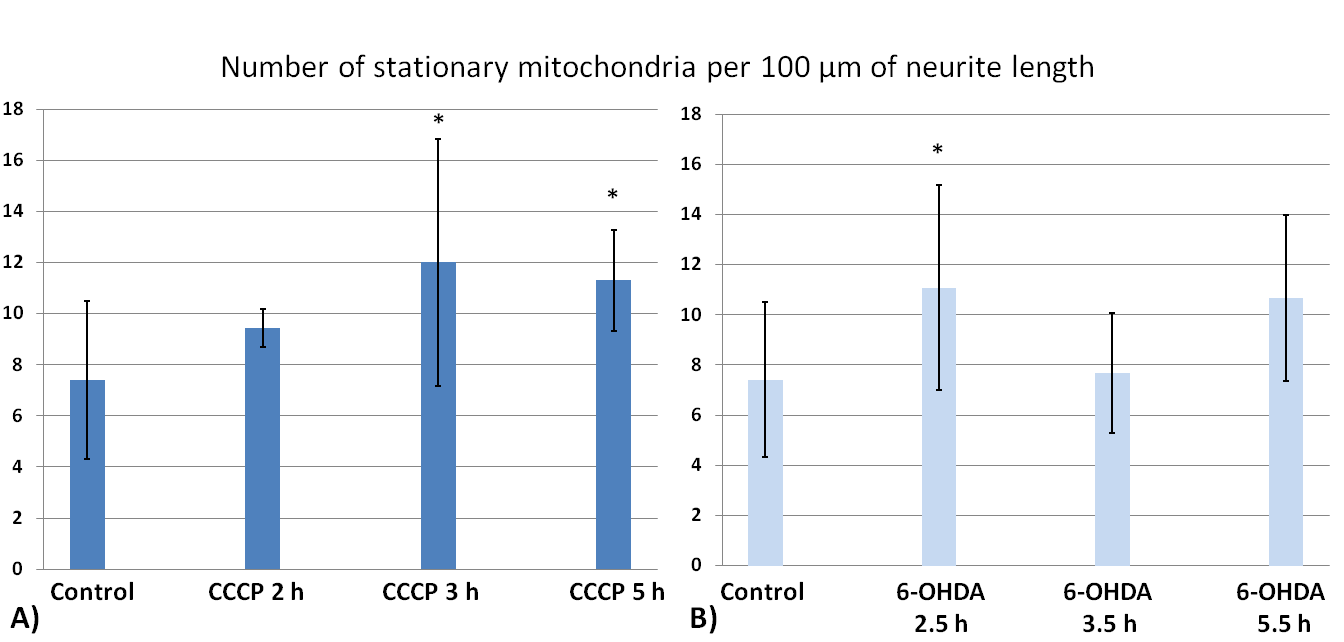

Supplement: Supplementary file 2 — Supplementary Fig. S2. The number of stationary mitochondria per 100 μm neurite length in cells treated with CCCP (A) or 6-OHDA (B). The number of stationary mitochondria was calculated from 20-40 μm in focus fragments of neurites and results were extrapolated. The statistical significance between treated cells and control is denoted by asterisk * - p < 0.05. ** - p < 0.005; *** - p < 0.0005 (TIFF 112 kb) [file 10571_2016_438_MOESM2_ESM.tif]
